# Comparison of outcomes and recurrence rates in patients undergoing single or double burr hole surgery for the treatment of chronic subdural hematoma in Bosnia and Herzegovina

**DOI:** 10.1016/j.bas.2024.102863

**Published:** 2024-07-04

**Authors:** Ibrahim Omerhodžić, Bekir Rovčanin, Ismar Ećo, Bakir Kudić, Salko Zahirović, Almir Džurlić, Adi Ahmetspahić, Mirza Pojskić

**Affiliations:** a Department of Neurosurgery, Clinical Center University of Sarajevo, Sarajevo, Bosnia and Herzegovina; b Faculty of Medicine, University of Sarajevo, Sarajevo, Bosnia and Herzegovina; c International Patient Services Department, Medipol Mega University Hospital Istanbul, Istanbul, Turkey; d Department of Neurosurgery, Philipps University Marburg, University Hospital Marburg, Germany

**Keywords:** Chronic subdural hematoma, Burr hole, Recurrence, Outcome

## Abstract

**Introduction:**

Chronic subdural hematoma (CSDH) is a prevalent condition commonly seen in elderly individuals, often requiring neurosurgical intervention.

**Research question:**

This study investigates patient characteristics and recurrence rates in CSDH patients treated with single or double burr hole surgery in Bosnia and Herzegovina. Methods: A retrospective study was conducted on patients treated for CSDH between January 2018 and December 2022. The diagnosis of CSDH was confirmed through preoperative CT or MRI of the brain. Patients underwent either single or double burr hole surgery based on the neurosurgeon's decision. Preoperative and postoperative brain CT scans, along with clinical outcomes, were analyzed.

**Results:**

A total of 87 patients were included in the study, with 102 burr hole surgeries performed. Among these, 49 patients received single burr hole surgery, while 53 patients underwent double burr hole surgery. Recurrence of CSDH occurred in 8.8% of cases, with no significant difference observed between the groups. Notably, single burr hole surgery demonstrated comparable efficacy to double burr hole surgery in terms of subdural reduction and occurrence of pneumocephalus, while exhibiting fewer complications and shorter hospitalization.

**Discussion and conclusions:**

burr hole surgery, whether performed as a single or double procedure, is an effective treatment option for CSDH, as it leads to positive outcomes in both radiological and clinical assessments of patients following surgery. The population of Bosnia and Herzegovina receives good neurosurgical care for CSDH.

## Highlights

• Burr hole surgery for chronic subdural hematoma (CSDH) is an effective intervention.
• Single burr hole surgery demonstrated comparable efficacy to double burr hole surgery.
• The population of Bosnia and Herzegovina receives good neurosurgical care for CSDH.

## 1 Introduction

Chronic subdural hematoma (CSDH) is a frequently encountered condition among elderly individuals and is commonly managed by neurosurgeons. The overall incidence of CSDH in the general population ranges from 8.2 to 17.6 cases per 100,000 people per year, with a significantly higher incidence observed in patients over the age of 80 years (Rauhala et al., 2020). The exact mortality rate of CSDH is challenging to ascertain due to the presence of multiple comorbidities in elderly patients. However, certain studies have reported a one-year mortality rate of 32% (Miranda et al., 2011). Recurrence is a prevalent complication following CSDH surgery, with varying rates reported in different studies, ranging from 0% to 30%. The risk factors associated with recurrence can be classified into three categories. Patient-related risk factors include age, alcoholism, diabetes, cerebral atrophy, seizure disorders, coagulopathy, history of ventriculoperitoneal shunt, use of anticoagulation or antiplatelet medication, poor clinical condition at the time of presentation, and a low Glasgow Coma Scale score. Radiological-related risk factors include inadequate brain re-expansion postoperatively, significant midline shift, the presence of substantial subdural air (pneumocephalus), highly dense hematoma on computed tomography (CT), thick hematoma on CT or magnetic resonance imaging (MRI), intraoperative identification of impaired brain re-expansion, and the presence of layered or multi-loculated hematomas. Surgical-related risk factors include inadequate postoperative drainage and the potential for dural vessel damage during surgery (Soleman et al., 2014; Desai et al., 2017; Kolias et al., 2014; Gurunathan, 2005; Chon et al., 2012). Of note, cerebral re-expansion after surgery has been identified as a crucial factor in the recurrence of chronic subdural hematoma, as demonstrated in a study of 295 patients (Han et al., 2022). Conversely, a meta-analysis encompassing 402 studies investigated 32 potential risk factors for CSDH recurrence, identifying 21 factors significantly associated with postoperative recurrence. However, only three factors garnered robust evidence: male gender, bilateral hematoma, and the absence of drainage (Zhu et al., 2021). Additionally, an intriguing study published in 2021 established blood type A as an independent risk factor for CSDH recurrence (Hirai et al., 2021).

However, the most crucial factors predicting the recurrence of CSDH remain unclear, with varying findings reported in different studies. Other studies have identified midline shift greater than 10 mm, brain atrophy, severe pneumocephalus, drainage volume exceeding 100 ml, and a hyperdense component within the hematoma as the strongest predictors of CSDH recurrence (Miah et al., 2021; Shen et al., 2019). The treatment options for CSDH encompass both non-surgical and surgical approaches. Non-surgical treatment is typically reserved for small asymptomatic CSDH cases. Surgical interventions are employed for large symptomatic CSDH, which may involve a simple burr hole procedure, or a craniotomy for organized CSDH of substantial size. Another potential treatment option is embolization of the middle meningeal artery, especially for CSDH cases associated with a higher risk of recurrence (Lee, 2019). Surgical management of CSDH is considered to be one of the less technically challenging procedures in the field of neurosurgery. The surgical approach and techniques used for CSDH treatment are generally similar between low-income and high-income countries (Laeke et al., 2023).

Burr hole surgery represents the prevailing surgical intervention for managing CSDH. This procedure involves the creation of either a single or double burr holes. Currently, there exists no definitive consensus regarding the selection between single or double burr holes. Such a decision primarily rests with the attending neurosurgeon, who evaluates the patient's clinical condition and radiological findings. Notably, there are researches findings that single burr hole procedures were linked to a considerably higher recurrence rate, prolonged hospitalization, and increased incidence of infections. Conversely, there have been other research findings suggesting that single burr hole surgery is equally effective as its double burr hole, rendering an additional burr hole unnecessary (Lee, 2019).

The aim of this study is to evaluate the characteristic of patients and recurrence of chronic subdural hematoma patients treated with single or double burr hole surgery in a middle-income country, Bosnia and Herzegovina.

## 2 Materials and methods

### 2.1 Patients and surgery

We carried out a retrospective study on patients who received treatment for chronic subdural hematoma (CSDH) at the Clinic of Neurosurgery, Clinical Center University of Sarajevo, from January 2018 to December 2022. The study was approved by the Institutional Review Board (protocol code 0901-2-82 from December 21, 2022). All patients underwent a preoperative CT or MRI scan of the brain to confirm the diagnosis of CSDH. Following preoperative preparation, the patients were treated with either a single or double burr hole surgery, depending on the preference of the neurosurgeon.

The burr hole surgery was performed with this technique. Linear incisions were made on the frontal or parietal areas of the scalp depending on the location of the CSDH and number of burr holes. The soft tissues were dissected, and a trepanation hole with a size of 14 mm was created in the bone. The dura mater was coagulated, and cruciform incisions were made, with coagulation of the dura edges. The chronic subdural hematoma, which had a “motor oil” appearance, was gradually drained. Subsequently, the subdural space was irrigated with warm physiological saline solution until it became colorless. Subgaleal closed suction drainage were placed and secured, and the skin is sutured with approximation of the edges. Any remaining air is evacuated. The drainage was removed 24–48h after surgery.

The patients were divided into two groups determined by the neurosurgeon's preference, considering the patients' clinical status, presence of comorbidities, location-specific factors, and radiological evidence. Patients afflicted with larger, hemispheric, and membrane-separated CSDH underwent a bilateral burr holes surgery procedure, while others received single burr hole surgery. One group underwent a single burr hole procedure, while the other group underwent a procedure with double burr holes. Postoperative CT scans were typically carried out within 24–72 h after surgery. Patients were administered either general anesthesia or local anesthesia, depending on their individual risk factors for general anesthesia. If a patient was taking anticoagulation or antithrombotic medication, these medications were discontinued a few days prior to surgery. In cases where emergency surgery was necessary, specific antidote therapy was administered immediately. The study excluded patients under the age of 18, those treated with craniotomy for CSDH, those with recurrent CSDH, known genetic coagulopathy, patients on hemodialysis, and patients with a history of previous cranial surgery or subdural empyema. All other patients were included in the study. All patients underwent a routine check-up one month after their CSDH surgery. Additionally, all patients were followed up for a period of one year after the CSDH treatment. If any patient developed a new onset of neurological symptoms during this one-year period, a brain CT scan was performed to confirm whether recurrence of CSDH had occurred. Radiological recurrence was defined as new reaccumulating in the subdural space on CT I brain scan, with characteristics of CSDH, larger than the previous finding on the postoperative CT. Recurrence was only considered when there was a need of reoperation of the patient.

### 2.2 Data collection

The following clinical and demographic data were recorded: sex, age, history of known head trauma, comorbidities (hypertension, diabetes mellitus, heart disease, and cerebral infarction), usage of anticoagulation drugs preoperatively, hospitalization days, Glasgow Coma Score, Markwalder grading score, bilateral or unilateral CSDH, recurrence, pre- and postoperative midline shift and volume of the subdural effusion and pneumocephalus, complications. Complications were defined as an intrahospital need for surgical intervention after the CSDH treatment, before the discharge. For the radiological and clinical evaluation, bilateral CSDH cases were analyzed separately.

### 2.3 CT measurements

On the first preoperative and first postoperative CT of the brain volume of CSDH and subdural space after drainage of the CSDH was measured. The postoperative subdural effusion volume and pneumocephalus volume was measured on the thickest point with the formula ((A x B x C)/2). Two authors individually in the first step selected an axial CT slide with the largest effusion or pneumocephalus volume. “A” was defined as the largest longitudinal length in millimeter (mm). “B” was defined as the maximum width of the effusion or pneumocephalus volume (in mm), and it was measured from the internal table of the skull to the brain cortex perpendicular to the A on the same slide. “C” was the height of effusion or pneumocephalus volume, measured as number of axial slices on which it was visible multiplied with the CT slice thickness. The estimated volume was a multiple of A, B, and C and then divided by 2 (Chan et al., 2022).

All patients were followed for one year after CSDH treatment. If they developed a neurological symptom a brain CT was performed to get the confirmation of recurrent CSDH. Recurrence was assumed only when there was a radiological confirmation of it.

All this data were compared between patients with single and double burr hole treatment for CSDH.

### 2.4 Statistical analysis

The results of the investigation were presented in tabular form utilizing the licensed Microsoft Word software package. Descriptive and inferential statistical analyses were conducted using IBM SPSS Statistics 28.0. For continuous quantitative variables, measures such as the mean ($\bar{x}$), median (Me), and standard deviation (SD) were calculated. Qualitative variables were described using frequencies (f) and relative frequencies (rf), and their statistical significance was determined through the Chi-square test.

The Shapiro-Wilk test was utilized to evaluate the normality of distribution. In cases of normally distributed continuous variables, the statistical significance of differences between two groups was assessed using the independent samples *t*-test. For variables which did not meet the assumptions of *t*-test, the non-parametric Mann-Whitney *U* test was applied.

Multivariable binary logistic regression was performed to identify the strength of associations with outcomes.

The initial selection of variables for the binary logistic regression model was guided by clinical knowledge and a thorough review of relevant literature. In the preliminary univariate analyses, all covariates with a p-value less than 0.05 were retained for inclusion in the binary logistic regression model.

All statistical tests were two-tailed, with a significance level set at α = 0.05. The null hypothesis was rejected when p < 0.05.

## 3 Results

**Fig. 1:**
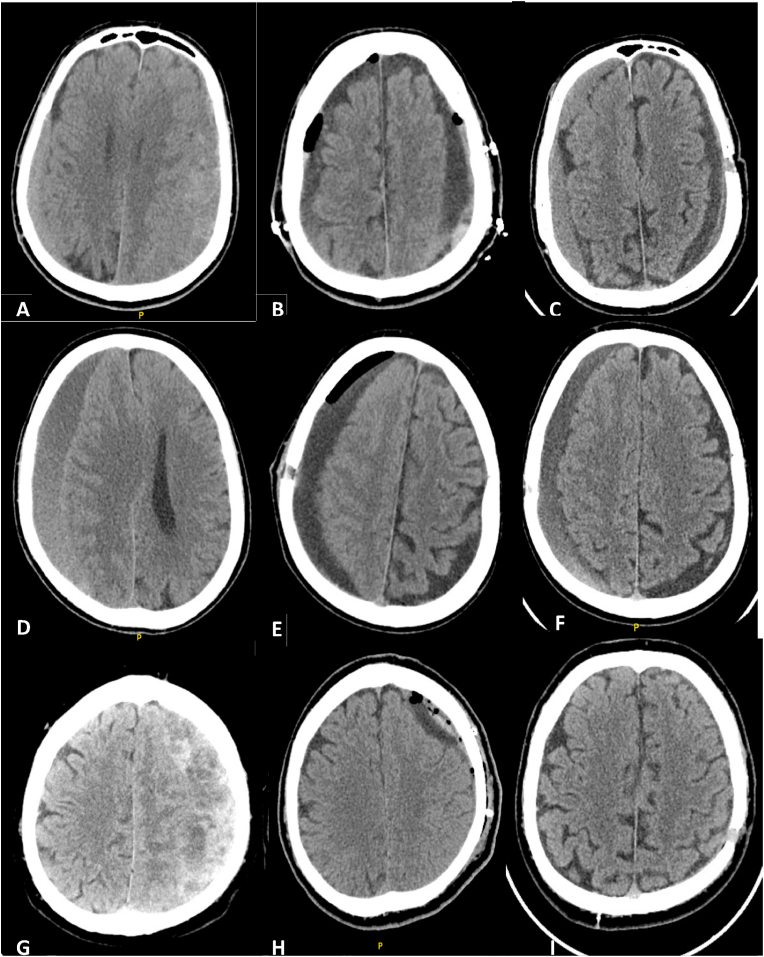
Difference between CT findings preoperatively and postoperatively for three patients. (A) Preoperative CT findings of an 83-year-old male patient with bilateral CSDH. (B) Postoperative CT findings after surgery with a single burr hole on the right and double burr holes on the left, showing good results.(C) CT findings 25 days post-surgery indicating recurrence of CSDH on the left side.(D) Preoperative CT findings of a 75-year-old male patient with unilateral right CSDH and a history of acetylsalicylic acid use.(E) Postoperative CT findings after treatment with a single burr hole surgery.(F) CT findings 36 days post-surgery revealing recurrence of CSDH.(G) Preoperative CT findings of a 60-year-old male patient with unilateral CSDH and membranes. (H) Postoperative CT findings after treatment with a double burr hole surgery. (I) CT findings 60 days post-surgery showing no signs of CSDH recurrence. CSDH- chronic subdural hematoma.

A total of 132 burr hole procedures for the treatment of chronic subdural hematoma were conducted at the Department of Neurosurgery at the University Clinical Centre in Sarajevo from 2018 to 2022. Of these, 30 patients were excluded due to non-compliance with inclusion criteria. A total of 87 patients were included in the study, 15 of them had bilateral and 72 of them had unilateral CSDH. When the bilateral CSDH were analyzed separately, every side of the bilateral CSDH was assumed as one case, 102 cases of CSDH were included, 49 were assigned to receive single burr hole and 53 to receive double burr holes (Fig. 1). The mean age of the study population was 75.0 ± 10.15 years, and there was a significant difference in age between the two study groups (p = 0.016), with those receiving single burr hole being significantly older. On admission, the mean Glasgow Coma Scale score was 12.69 ± 3.34, with no significant differences between the two study groups. The presence of comorbidities, including cardiovascular insult, arterial hypertension, cardiovascular diseases, trauma, use of anticoagulation or antiaggregating therapy did not significantly differ between the two study groups, where 7 patients used warfarin, one patient novel oral anticoagulants (NOAC) and 14 patients used acetylsalicylic acid. Patient characteristics, overall and stratified by groups are given in Table 1.

**Table 1:** Comparison of preoperative findings between patient groups with single and double burr holes.

Variable		Total	Burr holes		p-value
			Single	double	
Recurrence	Yes, n (%)	9 (8.8)	6	3	0.241^a^
	No, n (%)	93 (91.2)	43	50	
Sex	Male, n (%)	81 (79.4)	38	43	0.655^b^
	Female, n (%)	21 (20.6)	11	10	
DM	Yes, n (%)	21 (20.6)	14	7	0.055^c^
	No, n (%)	81 (79.4)	35	46	
Hypertension	Yes, n (%)	55 (53.9)	31	24	0.069^d^
	No, n (%)	47 (46.1)	18	29	
Stroke	Yes, n (%)	11 (10.8)	6	5	0.65^e^
	No, n (%)	91 (89.2)	43	48	
Cardiovascular diseases	Yes, n (%)	22 (21.6)	11	11	0.84^f^
	No, n (%)	80 (78.4)	38	42	
Other comorbidities	Yes, n (%)	32 (31.7)	21	11	**0.019**^g^
	No, n (%)	69 (68.3)	28	41	
Trauma	Yes, n (%)	23 (22.5)	12	11	0.65^h^
	No, n (%)	79 (77.5)	37	42	
Anticoagulation or antiaggregating therapy	Yes, n (%)	22 (31.4)	13	9	0.916^i^
	No, n (%)	48 (68.6)	29	19	
Age, mean ± SD		75.0 ± 10.15	76.67 ± 10.18	71.21 ± 12-25	**0.016**
GCS, mean ± SD		12.69 ± 3.34	12.88 ± 3.29	12.51 ± 3.41	0.581
MGWS, mean ± SD		1.93 ± 0.97	1.86 ± 0.068	2.00 ± 1.18	0.459
Subdural volume (cm^3^), mean ± SD		207.93 ± 81.47	119.07 ± 68.06	153.12 ± 64.06	0.282
Midline shift (mm), mean ± SD		7.67 ± 0.50	5.90 ± 4.59	8.34 ± 5.36	**0.016**

Bold: p < 0.05; IQR: interquartile range; SD: standard deviation; DM: diabetes; GCS- Glasgow coma scale; MGWS- Markwalder Grading Scale.

a χ2 = 1.37.

b χ2 = 0.20.

c χ2 = 3.68.

d χ2 = 3.31.

e χ2 = 0.21.

f χ2 = 0.043.

g χ2 = 5.49.

h χ2 = 0.203.

i χ2 = 0.011.

There was no difference between these two groups regarding recurrence of the CSDH (p = 0.241), and overall recurrence rate was 8.8%. In the group of the bilateral CSDH, two patients had recurrence of the CSDH. Patients in double burr hole group had significantly longer hospital stay (p = 0.009), as well as greater preoperative volume of the CSDH. Postoperative subdural space and midline shift were significantly higher (p = 0.003, and p = 0.024, respectively) in double burr hole group compared to the single burr hole group. Complications during hospital stay occurred more often in the double burr hole group as well (p = 0.026) (Table 1, Table 2).

**Table 2:** Comparison of radiological and postoperative findings between patient groups with single and double burr holes.

Variable		Total	Burr holes		p-value
			Single	Double	
Complications	Yes, n (%)	5 (5.0)	0	5	**0.026**^a^
	No, n (%)	96 (95.0)	49	47	
Hospitalization (days), mean ± SD		8.0 ± 2.0	5.85 ± 2.28	7.64 ± 3.91	**0.009**
Subdural volume postoperative (cm^3^), mean ± SD		71.47 ± 48.95	52.33 ± 42.05	58.99 ± 32.13	0.375
Subdural volume difference (cm^3^), median (IQR)		196.9 (105.05; 198.10)	56.62 (27.44; 92.25)	78.70 (55.07; 118.33)	**0.003**
Pneumocephalus volume (cm^3^), median (IQR)		1.5 (1.5; 2.05)	12.45 (0.6; 38.03)	7.75 (2.0; 29.23)	0.617
Midline shift difference (mm), mean ± SD		4.77 ± 3.02	3.05 ± 3.12	4.74 ± 3.77	**0.024**

Bold: p < 0.05; IQR: interquartile range; SD: standard deviation.

a χ2 = 4.96.

There was no statistically significant difference between characteristics of patients with recurrence and without recurrence. In the binary logistic regression analysis, only one independent predictor was identified as significant for complications, preoperative GCS (Glasgow Coma Scale). Preoperative GCS was negatively associated with an OR of 0.698 (95% CI: 0.53–0.92, p = 0.011), indicating that higher GCS scores were associated with a reduced risk of complications. The overall model was statistically significant (χ2 = 21.84, p < 0.0001, with R2 of 0.622) and accurately classifying 96.6% of cases (Table 3).

**Table 3:** Binary logistic regression analysis of independent predictors for complications.

Independent predictors	Coefficient (B)	Odds Ratio (95% CI)	p-value
Hospitalization	0.302	1.35 (0.98–1.88)	0.071
Preoperative GCS	−0.436	0.65 (0.46–0.91)	0.013
No of burr holes	−19.06	–	0.997

The model was not statistically significant χ2 = 21.84, p < 0.0001; it explained 62.2% (Nagelkerke R2) of the variance and correctly classified 96.6% of cases; CI: Confidence Interval; GCS: Glasgow Coma Scale.

## 4 Discussion

The overall recurrence rate of patients with CSDH in Bosnia and Herzegovina is 8.8%. We have demonstrated that performing a single burr hole surgery for chronic subdural hematoma does not lead to a different rate of recurrence compared to double burr hole surgeries. The results of this study suggest that burr hole surgery, whether performed as a single or double procedure, is an effective treatment option for chronic subdural hematoma (CSDH), as it leads to positive outcomes in both radiological and clinical assessments of patients following surgery. However, patients undergoing double burr hole surgeries had a higher incidence of complications, what can be attributed to a possible selection bias. Notably, the preoperative Glasgow Coma Scale was found to be an independent predictor of these complications.

According to global estimates, CSDH affects between 1.72 and 20.6 individuals per 100,000 people annually, with a notable increase in occurrence among older adults. A recent meta-analysis, comprising a total of 7337 patients with CSDH, demonstrated the utilization of various surgical techniques, including twist drill craniotomy in 15 studies, single burr hole craniotomy in 30 studies, double burr hole craniotomy in 21 studies, mini-craniotomy in 13 studies, and traditional craniotomy in a single study (Qiu et al., 2023). Despite advances in our understanding of the pathophysiology of chronic subdural hematomas, treatment options, and the utilization of standard surgery and endovascular embolization, the issue of recurrence still persists. Recurrence of chronic subdural hematomas continues to pose significant medical, economic, and social challenges. The reported global recurrence rate after CSDH surgery ranges from 5 to 30%, in our study 8.8% (Henry et al., 2022; Laeke et al., 2023). Chronic subdural hematoma surgery is a relatively uncommon neurosurgical procedure that, when executed with precision, leads to favorable clinical outcomes in both low and high-income countries (Laeke et al., 2023). There in a debate about risk factors for recurrence of CSDH, one of the reported possible risk factors is the number of burr holes placed during the surgery (Cofano et al., 2020). The pathophysiological mechanism which explains the recurrence include microvascular bleedings in a persistent cavity without compression to the blood vessels, but there is also an inflammatory reaction which contributes to the recurrence of the CSDH (Belkhair and Pickett, 2013; Jang et al., 2020). Despite there is more knowledge and more studies regarding the best treatment for CSDH there are not clear guidelines when to perform single or double burr holes in achieving best postoperative results. In our study there was no statistically significant difference between recurrence after single or double burr holes surgery for CSDH. We conducted a literature review, encompassing published meta-analyses from the past decade, to investigate the association between the number of burr holes employed during chronic subdural hematoma (CSDH) surgery and its influence on recurrence rates (Table 4).

**Table 4:** Published meta-analyses in last 10 years investigate the association between the number of burr holes employed during chronic subdural hematoma surgery and its influence on recurrence rates.

Authors	Year	Number of patients	Number of studies	Study design	Conclusion
Al-Salihi et al., 2023	2023	1135	11	RCTs and non-RCTs	DBHC seems to be the best modality for CSDH compared with sBHC and TDC. It showed significantly less recurrence and reoperation rates compared with TDC. On the other hand, DBHC showed no significant difference with the other comparators regarding complication, mortality, and cure rates in addition to the hospitalization duration.
Qiu et al. (2023)	2023	7337	38	RCTs, prospective study, or retrospective study	DBHC may be the most effective surgical treatment for CSDH based on the low recurrence and reoperation rates, although all examined techniques were relatively safe.
Henry et al. (2022)	2022	103,645	455	Studies comparing any 2 procedures	Recurrence after evacuation occurs in approximately 10% of CSDH, and the various surgical interventions are approximately equivalent.
Zhu et al. (2021)	2021	347–11889	402	Randomized, prospective, retrospective, and overall observational studies	Results showed that single- and double-hole as well as twist-drill surgeries did not affect the development of recurrence.
Wan et al. (2019)	2019	1723	12	Retrospective observational trial or randomized controlled trial	There are no significant differences in recurrence rate, complication rate, and morbidity between SBHC and DBHC in the treatment of patients with CSDH.
Belkhair and Pickett, 2013	2013	713	5	Observational retrospective cohort studies	SBHC is as good as DBHC in evacuating chronic subdural hematoma and is not associated with a higher revision rate compared to DBHC.

RCTs- Randomized Controled Trials; CSDH-chronic subdural heamathoma; SBHC- single burr-hole craniostomy, DBHC-double burr-hole craniostomy, TDC- twist-drill craniostomy.

The findings of these studies exhibit substantial heterogeneity, with some reporting that single burr hole surgery is good same as double burr hole surgery CSDH, while others advocate for double burr hole surgery (Jang et al., 2020; Taussky et al., 2008; Al-Salihi et al., 2023; Qiu et al., 2023; Wan et al., 2019). Furthermore, certain studies propose that the choice of burr hole quantity has no significant impact on the recurrence rate of CSDH (Zhu et al., 2021; Belkhair and Pickett, 2013; Wan et al., 2019; Henry et al., 2022). An umbrella study of systematic reviews and meta-analyses which included 32 risk factors did not find that the number of burr holes is a risk factor (Zhu et al., 2021).

It is well known that burr hole craniotomy in treatment of CSDH has better outcomes and less complication rate then craniotomy. In a study which included 1003 patients they concluded that that mini craniotomy was significantly associated with medical complications and serious surgical postoperative complications than burr hole craniotomy (Zolfaghari et al., 2021). A meta-analysis published in 2019, which included 12 studies has shown that no significant differences in recurrence rate, complication rate, and morbidity was between single and double burr hole craniotomy (Wan et al., 2019). In our study there was a statistical difference between complication rate between single and double burr hole craniotomy group, where patients in double burr hole craniotomy had higher rate (p = 0.026). But in the binary logistic regression there was no any statistical proof that the number of burr holes is a predictor of complications, with a high p value almost 1. These results are supporting a study performed by Belkhair and Picket published in 2013, they concluded that number of burr holes is not associated with the postoperative clinical outcome or with the complications rate (Belkhair and Pickett, 2013). We found in this study that preoperative Glasgow coma score is a negative independent predictive factor for after surgery complications (OR: 0.65; 95% CI: 0.46–0.91; P = 0.013). Preoperative Glasgow coma score on admission has been considered as risk factor of CSDH recurrence in previous studies (Kang et al., 2007; Amirjamshidi et al., 2007).

One of the most common risk factors for recurrence of CSDH is a poor brain expansion after surgery. In a study which included 295 patients treated for unilateral CSDH authors have found that the key factors for predicting unilateral CSDH recurrence was cerebral re-expansion rate (≤40%) at postoperative days 7–9 (p < 0.001) (Han et al., 2022). Also, there are more studies with a concussion that poor brain expansion after surgery is a risk factor for CSDH recurrence (Lee, 2019). In a study published in Scientific Reports which included 291 patients treated for CSDH the found that subdural space, or depressed brain volume >50 cm3 shown in CT scans 7 days after the treatment is an independent predictor of recurrence of CSDH (Jang et al., 2020). We found in our study that patients in both groups treated with single or double burr hole craniotomy had in first 48–72 h subdural volume 52–59 cm3. Where patients in double burr hole had statistically significant huger subdural volume. However, this also can be explained that most neurosurgeons preferred more double burr holes craniotomy for patients with higher preoperative subdural hematoma and higher midline structures shift. The subdural volume difference preoperative and postoperative was statistically significant different between two groups, where the difference was bigger in double burr hole craniotomy group. Also, the postoperative values of midline structures shift in higher in double burr holes craniotomy group, but less than 5 mm (Table 1, Table 2). Postoperative midline structures shift is not found to be a factor for CSDH recurrence (Jang et al., 2020). Also, in our study value of in midline structures shift was not different between recurrent and non-recurrent group. Patients in the double burr holes surgery group in our study are significant younger 71.21 ± 12.25 years. We assume that our colleagues performed single burr hole craniotomy in older patients because the older patients are more fragile and to shorter the time of surgery and anesthesia. Age of the patients is considered as factor of recurrence of CSDH in some studies, but in other studies they do not support this (Ahmed et al., 2021). These radiological and bassline characteristics of patients are influencing a neurosurgeon's decision how many burr hole craniotomies to perform in our study. The patients preoperative GCS was without difference between groups, and it seems that size of the CSDH and patients ages are mostly influencing this decision, in leak of established clear guidelines for treatment of CSDH. Performing single burr hole surgery is less invasive, with shorter surgery time than double burr hole surgery. Some studies have reported that single burr hole surgery is not effective like double burr hole surgery, because there is not enough irrigation of the subdural space, especially in separated hematomas (Stanisic et al., 2005; Han et al., 2009). On the other hand, a study conducted by Yamamato H. et al. Which included 105 patients concluded that single burr hole irrigation is sufficient to resolve CSDH, also in cases with in multiple cavities (Yamamoto et al., 2003). A recent study confirmed that single burr hole surgery with drainage is sufficient approach to achieve a good surgical outcome with a low complication rate (Oishi et al., 2001).

Postoperative pneumocephalus is reported is some studies as an independent predictor of recurrence. It was assumed that air in the subdural space restricts the brain expansion and obtains the cavity, which can lead to recurrence of CSDH (Huang et al., 2020). Another study published in 2020 concluded that postoperative pneumocephalus at certain range has no effect on the prognosis of patients (Sánchez Fernández et al., 2022). We have found in our study that there is no statistical difference between single or double burr hole surgery group regarding the postoperative pneumocephalus volume.

Hospitalization duration is a one of huge problems for the health system and for the patient. Sánchez Fernández C. et al. Also reported that the hospital stays of patients treated with one burr hole was shorter, but without any statistically significant (Sánchez Fernández et al., 2022). In our study we concluded also that the patients treated with one burr hole have a statistically significant shorter hospital stay.

The embolization of the middle meningeal artery has emerged as a promising intervention for the management of CSDH. This technique is being increasingly employed both as an adjunctive therapy in conjunction with surgical evacuation, as well as a standalone approach for CSDH treatment. Its effectiveness, especially in recurrent cases, has garnered attention. Analysis of 12 randomized clinical trials, along with some non-randomized studies, has demonstrated that the embolization of the middle meningeal artery is a safe and effective method for managing CSDH. It has been found to be successful as a primary treatment, in recurrent cases, and as postoperative prophylaxis. However, these studies exhibit significant heterogeneity in their data, warranting further investigations before making a definitive recommendation (A type) for its widespread implementation. Within the neurosurgical community, there remains considerable uncertainty surrounding the indications and patient selection criteria for this innovative approach to CSDH treatment. Hence, additional prospective clinical trials are needed to gain a deeper understanding of the optimal indication and long-term benefits of embolization of the middle meningeal artery (Lee, 2019; Henry et al., 2022; Solou et al., 2022).

To our knowledge, there is a limited number of studies on CSDH in our region. However, we did find one notable study conducted in our region, spanning Serbia and Croatia, from 2018 to 2019. The study investigated patients with CSDH and found that approximately 20.6% of patients had bilateral CSDH, which is comparable to the near-similar prevalence of 17% observed in our own study (Koruga et al., 2024).

### 4.1 Study limitations

This study has some limitations that may impact the accuracy of the results. The retrospective design is a significant potential issue, as recorded data may not be entirely accurate. Additionally, the small sample size and single-center design, which only included one institution, may not be representative of the entire region of Bosnia and Herzegovina. Furthermore, the surgeon's preference played a major role in deciding how many burr holes to perform, which could have influenced the observed outcomes. Specifically, it appears that neurosurgeons may have performed double burr hole surgery more frequently in cases with more severe hematomas, which may explain the worse outcomes in terms of length of stay and complications observed in this group. Unfortunately, there is no information available on the use of corticosteroids and their potential impact on the results. Therefore, we believe that a larger, multicenter, prospective, and randomized study involving all neurosurgical centers in Bosnia and Herzegovina is needed to validate our findings and provide national guidelines for the treatment of CSDH.

## 5 Conclusions

In this study we did not find a difference between recurrence rate of chronic subdural hematoma before and after the surgery, in patients groups treated with single or double burr holes surgery. The results of this study suggest that burr hole surgery, whether performed as a single or double procedure, is an effective treatment option for chronic subdural hematoma, as it leads to positive outcomes in both radiological and clinical assessments of patients following surgery. These results support the thesis that the population of Bosnia and Herzegovina, as a middle-income country, receives good neurosurgical care for CSDH, as evident by the low recurrence rate of 8.8%.

## Author contributions

Conceptualization, I.O. and B.R.; methodology, M.P. software, B.K.; validation, I.O., B.R. and A. DŽ.; formal analysis, I.E. and B.K.; investigation, S.Z.; resources, A.A.; data curation, I.E.; writing—original draft preparation, B.R.; writing—review and editing, A. DŽ.; visualization, A.A.; supervision, M.P.; project administration, S.Z. All authors have read and agreed to the published version of the manuscript.

## Funding

This research received no external funding.

## Conflicts of interest

The authors declare no conflicts of interest.

## Declaration of competing interest

The authors declare that they have no known competing financial interests or personal relationships that could have appeared to influence the work reported in this paper.
